# Integrated electrophysiological and payload-release assessment of neuronal responses to antibody-drug conjugates in human iPSC-derived sensory neurons: implications for *in vitro* neurotoxicity evaluation

**DOI:** 10.3389/ftox.2026.1855330

**Published:** 2026-09-24

**Authors:** Taeko Kuroda, Xiaobo Han, Toshiki Mochizuki, Yuto Ishibashi, Aya Goto, Yuki Seki, Norimasa Miyamoto, Takashi Yoshinaga, Shoji Asakura, Ikuro Suzuki

**Affiliations:** 1 Department of Electronics, Graduate School of Engineering, Tohoku Institute of Technology, Sendai, Japan; 2 Global DMPK, Biopharmaceutical Assessment, DHBL, Eisai Co., Ltd., Tsukuba, Japan; 3 Global Drug Safety, Biopharmaceutical Assessment, DHBL, Eisai Co., Ltd., Tsukuba, Japan; 4 Integrated ADMET Science, Biopharmaceutical Assessment, DHBL, Eisai Co., Ltd., Tsukuba, Japan; 5 Advanced Biosignal Safety Assessment, Biopharmaceutical Assessment, DHBL, Eisai Co., Ltd., Tsukuba, Japan

**Keywords:** antibody-drug conjugates, human iPSC-derived sensory neurons, microelectrode array, neurotoxicity, payload release, peripheral neuropathy

## Abstract

Antibody-drug conjugates (ADCs) enable tumor-specific drug delivery; however, target antigen-independent adverse events, including peripheral neuropathy, remain a significant clinical concern. The mechanisms underlying ADC-induced neurotoxicity, particularly the relationship between payload release and alterations in neuronal function, are not yet fully understood. In this study, we established an analytical platform that integrates microelectrode array (MEA) recordings using human induced pluripotent stem cell (iPSC)-derived sensory neurons with quantitative measurement of payload levels detected after ADC exposure under culture conditions, enabling functional evaluation of the direct effects of ADCs and their payloads on neuronal activity. The microtubule inhibitors monomethyl auristatin E (MMAE) and Lys-SMCC-DM1, when administered as free payloads, suppressed neuronal activity, as indicated by reduced firing rates, decreased spike amplitudes, together with neurite fragmentation. In contrast, their corresponding ADCs, brentuximab vedotin and trastuzumab emtansine, showed limited detectable effects on neuronal activity under the present experimental conditions. Conversely, the topoisomerase I inhibitor SN-38 enhanced neuronal activity, as evidenced by increased firing rates and spike amplitudes without marked morphological disruption. Similar functional changes were observed with sacituzumab govitecan. Furthermore, quantitative analysis revealed substantially higher measured payload levels at the 168-h endpoint following sacituzumab govitecan exposure than following brentuximab vedotin or trastuzumab emtansine exposure under the present experimental conditions. These findings suggest that the neuronal functional responses observed following ADC exposure may be associated with differences in measured payload levels and are consistent with the possibility of target antigen-independent effects. The integrated platform developed in this study enables quantitative assessment of neuronal functional responses to ADCs and payloads and may serve as a complementary tool for *in vitro* neurotoxicity evaluation during ADC design and development.

## 1 Introduction

Antibody-drug conjugates (ADCs) have rapidly emerged as a novel class of anticancer therapeutics, integrating the target specificity of monoclonal antibodies with the potent cytotoxicity of small-molecule payloads (Beck et al., 2017; Chau et al., 2019). To date, numerous ADCs have been successfully translated into clinical practice and are increasingly established as highly effective treatment modalities (Drago et al., 2021). However, adverse events, including dose-limiting toxicities (DLTs), remain a significant clinical challenge (Saber and Leighton, 2015). While the toxicological profile of an ADC depends on its specific payload, peripheral neuropathy and hematologic toxicity frequently represent the primary factors limiting treatment continuation (Saber and Leighton, 2015). Among these, peripheral neuropathy is particularly problematic; it often develops in a dose-dependent and cumulative manner and may persist long after treatment discontinuation (Cavaletti and Marmiroli, 2015; Staff et al., 2017). Despite its clinical prevalence, effective preventive or therapeutic strategies have yet to be established (Loprinzi et al., 2020). Notably, ADCs incorporating microtubule inhibitors such as monomethyl auristatin E (MMAE) and maytansinoid DM1 are frequently associated with a high incidence of peripheral neuropathy (Perez et al., 2014; Suri et al., 2019). Elucidating the functional mechanisms underlying this toxicity is therefore a critical priority, as it directly impacts both the sustainability of treatment and the patient’s quality of life.

The toxicity profile of ADCs is influenced not only by the antibody component but also by linker stability and the pharmacological properties of the payload (Donaghy, 2016; Beck et al., 2017; Chau et al., 2019; Chis et al., 2024; Ruan et al., 2024). Microtubule inhibitors such as MMAE and DM1 disrupt axonal transport by interfering with microtubule dynamics and exhibit high sensitivity in the peripheral nervous system (Jordan and Wilson, 2004; Staff et al., 2017). In contrast, the topoisomerase I inhibitor SN-38 is primarily associated with hematologic and gastrointestinal toxicities and has been considered to have relatively limited direct effects on peripheral neurons, although the occurrence of neuropathy cannot be excluded (Mathijssen et al., 2001; Mathijssen et al., 2002; Saber and Leighton, 2015). A comprehensive analysis of clinical trials and relevant FDA review documents indicate that the incidence of chemotherapy-induced peripheral neuropathy (CIPN) ranged from 23% to 52% for ADCs containing MMAE (e.g., brentuximab vedotin) or DM1 (trastuzumab emtansine), while sacituzumab govitecan, which contains SN-38 as its payload, was also associated with peripheral neuropathy in approximately 20% of cases (U.S. Food and Drug Administration, 2011a; U.S. Food and Drug Administration, 2011b; U.S. Food and Drug Administration, 2013a; U.S. Food and Drug Administration, 2013b; U.S. Food and Drug Administration, 2020; Kesireddy et al., 2024). These findings suggest that ADC-induced neurotoxicity cannot be explained solely by the pharmacological properties of the payload but likely involves multiple factors, including antigen-antibody interactions, linker characteristics, and payload release dynamics (Beck et al., 2017; Chau et al., 2019). Therefore, a functional evaluation framework that integrates payload mechanism of action with release behavior, including target antigen-independent effects, is needed to better understand peripheral neuronal activity.

Traditional toxicity assessments have largely relied on endpoint-based measures such as cell viability, morphological changes, and organ toxicity in *in vivo* models (Tarasiuk et al., 2024). However, these approaches may not adequately capture early functional alterations in neuronal activity underlying peripheral neuropathy (Kramer et al., 2020; Herr, 2021). In general, electrophysiological techniques, including patch-clamp recording, calcium imaging, and microelectrode array (MEA)-based extracellular recordings, are widely used to assess neuronal function (Jones et al., 2011; Buccino et al., 2022). Among these, MEA provides a non-invasive platform with high temporal resolution, enabling high-throughput and sensitive detection of changes in electrophysiological activity in cultured neurons (Novellino et al., 2011; Obien et al., 2015). While MEA-based systems have been increasingly applied in neurotoxicity assessment, their utility for evaluating peripheral neurotoxicity induced by ADCs and their payloads remains unclear (McConnell et al., 2012; Hondebrink et al., 2016; Kang et al., 2022). Direct comparison of free payloads and their corresponding ADCs using electrophysiological readouts may provide a useful approach for quantitatively characterizing functional differences.

In this study, we used human induced pluripotent stem cell (iPSC)-derived sensory neurons as an *in vitro* representative model of peripheral neurons to evaluate the effects of clinically relevant payloads (MMAE, DM1, and SN-38) and their corresponding ADCs under the experimental conditions used in this study. By integrating electrophysiological measurements (MEA), structural analyses (morphology and resistance), and quantitative assessment of payload release, we systematically evaluated the impact of ADCs on peripheral neuronal activity from a functional perspective. The findings of this study could contribute to a better understanding of ADC-associated neuronal functional changes, including those observed under conditions with no detectable target antigen expression, and provide a basis for improving ADC safety through optimized linker design and payload selection.

## 2 Methods and materials

### 2.1 Immunohistochemistry in human samples

Commercially available de-identified formalin-fixed paraffin-embedded human dorsal root ganglion (DRG) sections (HP-240; Zyagen, San Diego, CA, United States) were utilized for immunohistochemical analysis. To assess the expression of specific antigens, sections were incubated with the following primary antibodies: anti-CD30 (ab252561), anti-Trop2 (ab214488), and anti-HER2 (ab16901), all purchased from Abcam (Cambridge, United Kingdom). Heat-induced epitope retrieval was performed using Bond Epitope Retrieval Solution 1 (ER1; AR9961, Leica Biosystems, Newcastle upon Tyne, United Kingdom). Immunoreactivity was visualized using the BOND Polymer Refine Detection system (DS9800, Leica Biosystems) with 3,3′-diaminobenzidine (DAB) as the chromogen, followed by hematoxylin counterstaining of the nuclei.

### 2.2 Culture of human iPSC-derived sensory neurons

Human iPSC-derived sensory neurons (RealDRGx™ Nociceptors; ANATOMIC, Minneapolis, MN, United States) were used in this study. According to a published transcriptomic analysis by the supplier, these cells exhibit characteristics similar to embryologically mature human sensory neurons after 3 weeks of culture, and non-sensory neuronal cell types, including glial cells, fibroblasts, and immune cells, were not identified in this cell model (Truong et al., 2024). The cells were seeded onto a 48-well MEA plate (M768-tMEA-48W; Axion BioSystems, Atlanta, GA, United States). Each MEA plate was pre-coated with poly-D-lysine (Sigma-Aldrich, St. Louis, MO, United States) 1 day prior to cell seeding. On the day of seeding, the plate was further coated with iMatrix-511 (Matrixome, Osaka, Japan) and incubated at 37 °C with 5% CO_2_ for at least 1 h. After removal of iMatrix-511, cells were seeded at a density of 4 × 10^4^ cells per well using a 3.4-mm cloning ring (IWAKI, Shizuoka, Japan). After confirming cell attachment under a microscope, the cloning ring was carefully removed, and culture medium was added to each well. Cells were maintained in Senso-MMx2 (ANATOMIC), with half of the medium replaced every 2–3 days.

### 2.3 Pharmacological tests

Pharmacological tests were conducted on human iPSC-derived sensory neurons after 3–4 weeks of culture, when stable spontaneous spiking activity was observed. Details of the test compounds are summarized in Table 1. The concentration ranges used in this study were selected based on reported IC50 values from published cancer cell-based cytotoxicity assays. Approximate median IC50 values were estimated for each ADC and corresponding payload, and test concentrations were selected on a logarithmic scale to cover a broad range encompassing these reference values. The literature-derived IC50 values are summarized in Supplementary Table S1. Each compound was reconstituted, stored, and handled according to the manufacturer’s instructions. Working solutions were prepared by diluting each compound in the respective vehicle (DMSO, sterile water, or physiological saline), as indicated in Table 1, and the final vehicle concentration in each well was adjusted to 0.1%, including vehicle control wells. Vehicle controls were prepared using the corresponding vehicle for each compound and were matched to the final vehicle concentration used in the treatment wells. MEA-derived resistance and spontaneous activity were measured before drug administration (0 h) and 2, 24, 72, and 168 h after administration (n ≥ 6). Cultures were maintained in medium containing the respective compounds throughout the recording period, with a final medium volume of 500 µL per well. After the 72-h measurement, half of the medium was replaced in all wells; 250 µL of medium was removed from each well and replaced with 250 µL of freshly prepared medium containing the corresponding compound at the same final concentration as the original treatment medium.

**TABLE 1:** List of test compounds.

Compound	Category	Supplier	Catalog/Product no.	Lot/Batch no.	Vehicle	Concentration range (ng/mL)
Brentuximab vedotin	ADC	Takeda	Adcetris	553,572	Sterile water	1, 10, 100, 1,000, 10,000
Trastuzumab emtansine	ADC	Chugai	Kadcyla	23J010Z	Sterile water	1, 10, 100, 1,000, 10,000
Sacituzumab govitecan	ADC	MedChemExpress	HY-132254	286,558	Physiological saline	10, 100, 1,000, 10,000, 100,000
Monomethyl auristatin E (MMAE)	Payload	MedChemExpress	HY-15162	115,595	DMSO	0.01, 0.1, 1, 10
Lys-SMCC-DM1	Payload	MedChemExpress	HY-101982	282,963	DMSO	1, 10, 100, 1,000
SN-38	Payload	MedChemExpress	HY-13704	268,112	DMSO	1, 10, 100, 1,000

Vehicle controls were prepared using the corresponding vehicle for each compound, and the final vehicle concentration in each well was adjusted to 0.1% across all treatment and vehicle control wells.

### 2.4 Extracellular recordings

MEA viability/resistance measurements and spontaneous field potentials were recorded at 37 °C with 5% CO_2_ using an MEA system (Maestro Pro; Axion Biosystems), and analyzed using AxIS Navigator software (Axion Biosystems) and MATLAB (MathWorks, Natick, MA, United States). Recordings were performed using 48-well MEA plates with 16 microelectrodes per well. After placement of the MEA plates, impedance-based MEA Viability measurements were performed using the default Neural + Viability configuration of AxIS Navigator. In this assay, a small electrical signal is delivered through the microelectrodes, and the reported parameter is resistance at 41.5 kHz. Resistance values were recorded for each electrode and expressed in kΩ. In the present analysis, the exported endpoint “Resistance - Avg (kΩ)” was used as the well-level resistance parameter. Spontaneous activity was then recorded for 15 min at a sampling rate of 12.5 kHz per channel. Spikes were detected when the extracellular signal exceeded ±5.3 standard deviation of baseline noise during quiescent periods. Pharmacological responses were evaluated based on spontaneous spiking activity, including spike rate, mean spike amplitude, and the coefficient of variation (CV) of spike amplitude. Electrodes exhibiting firing rates ≥5 spikes/min were defined as active electrodes. The exclusion criterion was defined as fewer than 9 active electrodes out of 16 before drug administration. However, no wells met the exclusion criteria, and all recorded wells were included in the final analysis. The same wells were tracked throughout the experiments. To distinguish compound-induced electrophysiological effects from time-dependent baseline changes, electrophysiological data were first normalized to the 0 h value within each treatment group. For Figure 1E, firing-rate data are presented after normalization to the corresponding 0-h value within each treatment group. For Figures 1F–H, 2B, 3A–D, and Supplementary Figure S2, electrophysiological and resistance data were first normalized to the corresponding 0-h value within each treatment group and then normalized to the concurrent vehicle control at each time point. For each condition, n = 6 wells were analyzed, obtained from 2 to 3 plates with three wells per condition per plate where applicable. An additional independent experiment was performed using a different neuronal lot to assess the reproducibility of the spike rate responses to MMAE and SN-38. In this independent experiment, n = 6 wells per condition were analyzed using two plates with three wells per condition per plate. The same wells were tracked longitudinally throughout each experiment.

**FIGURE 1:**
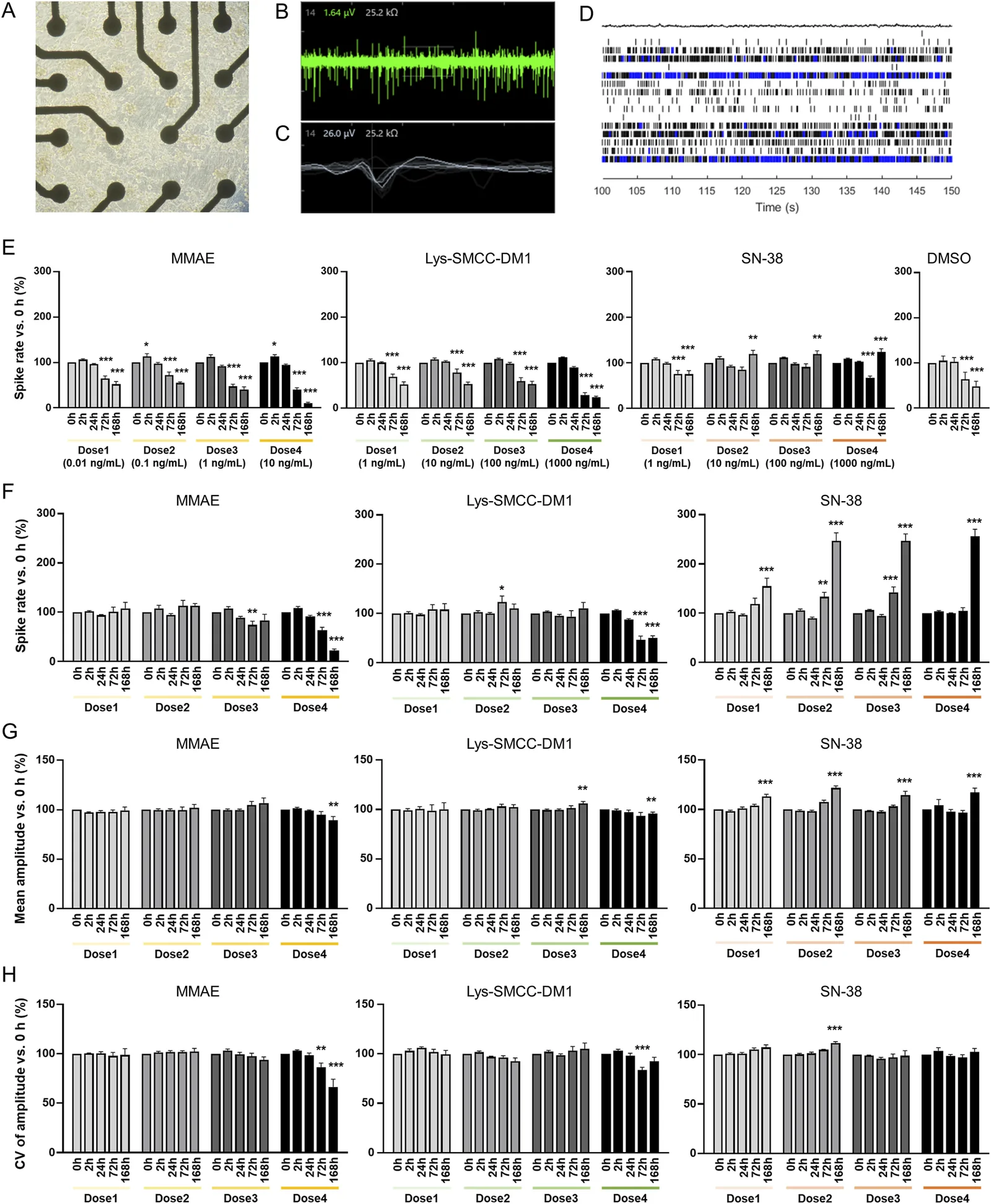
Effects of payloads on neuronal electrical activity in hiPSC-derived sensory neurons. **(A)** Phase-contrast image of hiPSC-derived sensory neurons cultured on a microelectrode array (MEA) chip for 4 weeks in vitro. **(B, C)** Representative extracellular action potential waveforms recorded from a single electrode. **(D)** Representative raster plot showing spontaneous firing activity over 50 s across all electrodes within a single well. **(E)** Time-course changes in spike firing frequency following treatment with MMAE, Lys-SMCC-DM1, SN-38, or vehicle control (DMSO). Data were normalized to the 0-h value within each treatment group. **(F–H)** Quantitative analysis of neuronal activity parameters first normalized to the corresponding 0-h value within each treatment group and then normalized to the corresponding vehicle control at each time point: spike firing frequency **(F)**, mean spike amplitude **(G)**, and coefficient of variation (CV) of spike amplitude** (H)**. Data were obtained from 15-min recordings (n = 6 wells per condition) at 0, 2, 24, 72, and 168 h posttreatment across four concentration conditions. Statistical analysis was performed using a linear mixed-effects model or generalized estimating equation model for repeated-measures data, followed by Dunnett-type adjusted post hoc comparisons versus the corresponding 0-h value within each dose group (*p < 0.05, **p < 0.01, ***p < 0.001). Data are presented as mean ± SEM.

**FIGURE 2:**
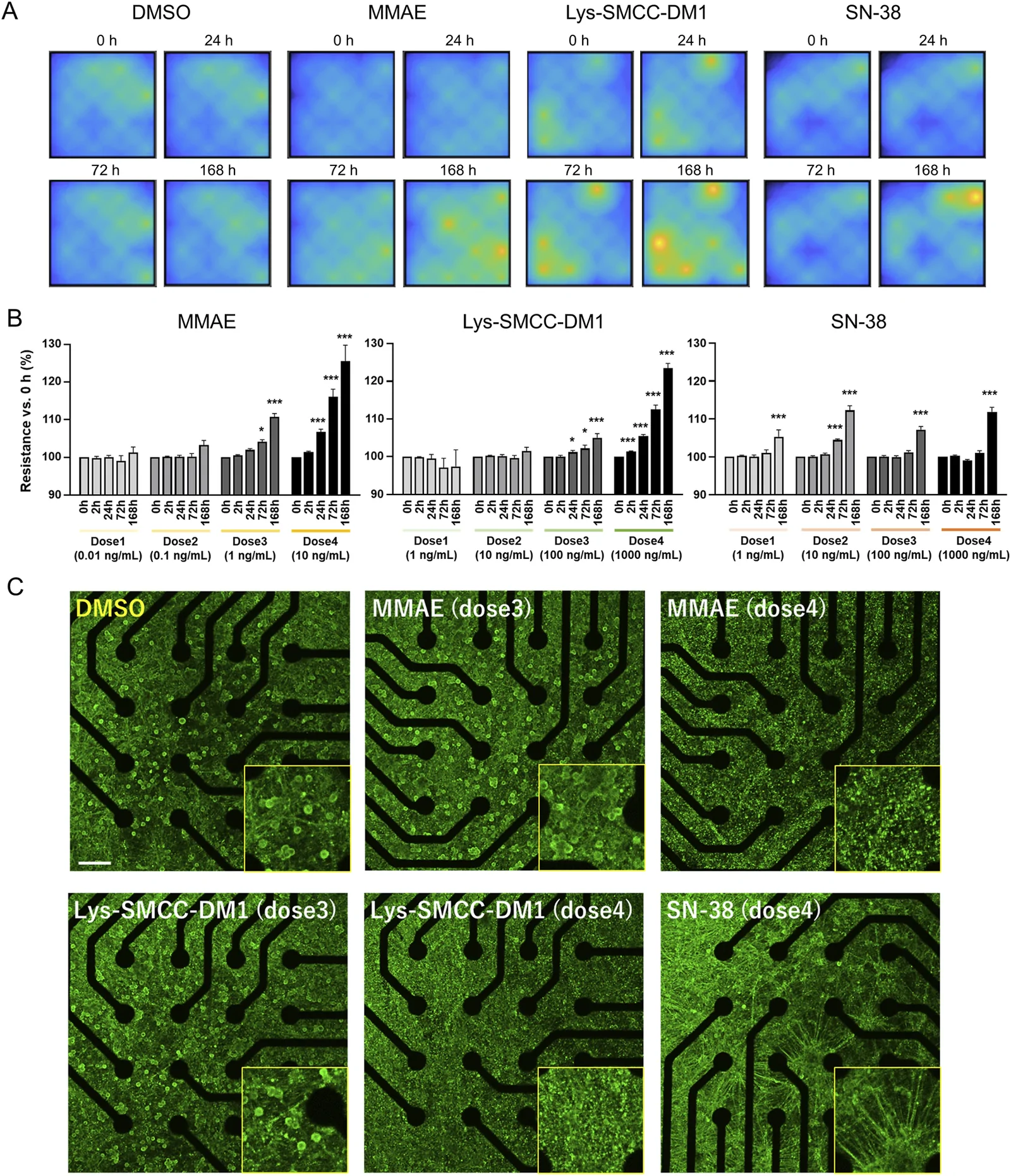
Effects of payloads on resistance and cellular morphology in hiPSC-derived sensory neurons. **(A)** Representative resistance heatmaps across the MEA electrode array obtained before and after treatment with MMAE, Lys-SMCC-DM1, SN-38, or vehicle control (DMSO). **(B)** Quantitative analysis of resistance changes over time, first normalized to the corresponding 0-h value within each treatment group and then normalized to the corresponding vehicle control at each time point (n = 6 wells per condition). **(C)** Representative immunocytochemical images of hiPSC-derived sensory neurons at 168 h post-treatment, stained for βIII-tubulin to visualize neuronal morphology. Images are shown for the DMSO control, MMAE at doses 3 and 4, Lys-SMCCDM1 at doses 3 and 4, and SN-38 at dose 4. Insets show enlarged views of representative regions. Scale bar = 200 μm. Statistical analysis was performed using a linear mixed-effects model or generalized estimating equation model for repeated-measures data, followed by Dunnett-type adjusted comparisons versus the corresponding 0-h value within each dose group (*p < 0.05, **p < 0.01, ***p < 0.001). Data are presented as mean ± SEM.

**FIGURE 3:**
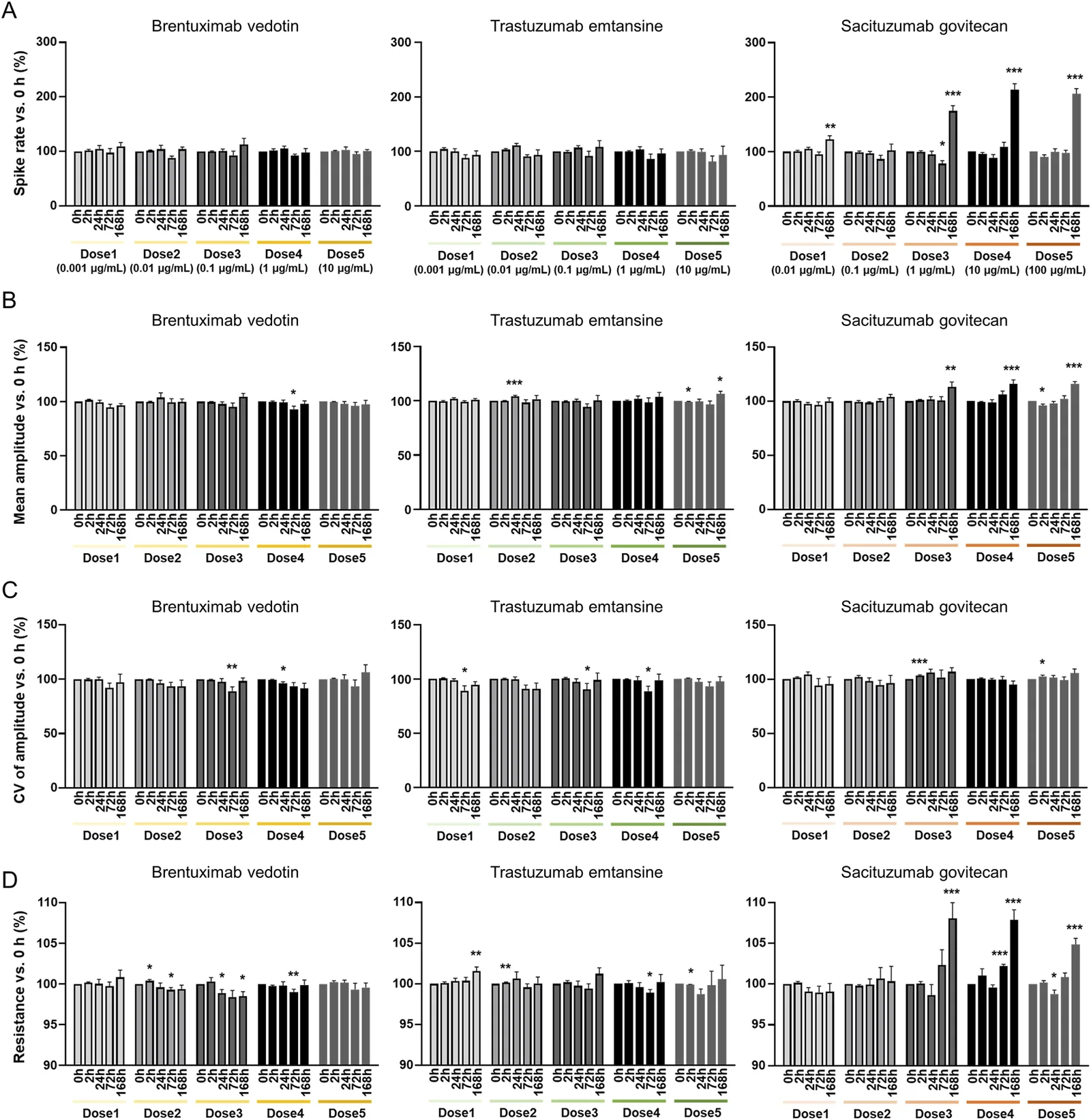
Effects of antibody-drug conjugates on neuronal electrical activity and resistance in hiPSC-derived sensory neurons. hiPSC-derived sensory neurons were treated with brentuximab vedotin, trastuzumab emtansine, or sacituzumab govitecan under five concentration conditions, and neuronal electrical activity and resistance were analyzed using MEA. **(A–C) **Quantitative analysis of neuronal activity parameters: spike firing frequency **(A)**, mean spike amplitude **(B)**, and coefficient of variation (CV) of spike amplitude **(C)**. **(D)** Time-dependent changes in resistance following ADC treatment. Data were first normalized to the 0-h value within each treatment group and then normalized to the corresponding vehicle control at each time point. Measurements were obtained from 15 min recordings at 0, 2, 24, 72, and 168 h post-treatment (n = 6 wells per condition). Statistical analysis was performed using a linear mixed-effects model or generalized estimating equation model for repeated-measures data, followed by Dunnett-type adjusted comparisons versus the corresponding 0-h value within each dose group (*p < 0.05, **p < 0.01, ***p < 0.001). Data are presented as mean ± SEM.

### 2.5 Immunocytochemistry

Human iPSC-derived sensory neurons were seeded onto 96-well glass-bottom plates (IWAKI) and fixed with 4% paraformaldehyde (PFA) prepared in phosphate-buffered saline (PBS). After washing with PBS, cells were permeabilized with 0.1% Triton X-100 in PBS at room temperature for 15 min and then blocked with 1% bovine serum albumin (BSA) in PBS at room temperature for 90 min. Cells were incubated overnight at 4 °C with primary antibodies against CD30, Trop2 and HER2 (same as those used for immunohistochemistry) diluted 1:100. After washing with PBS, cells were incubated with Alexa Fluor™ Plus 594-conjugated secondary antibodies (Thermo Fisher Scientific, Waltham, MA, United States) for 60 min at room temperature. Nuclei were counterstained with DAPI (40,043, Biotium, Fremont, CA, United States) diluted 1:1,000 for 5 min.

For morphologic evaluation, cell cultures after MEA measurements at 168 h were fixed with 4% PFA in PBS for 10 min. After fixation, samples were permeabilized with 0.2% Triton X-100 in PBS for 5 min and subsequently blocked with 5% fetal bovine serum (FBS) and 0.05% Triton X-100 in PBS at 4 °C for 1 h. The cultures were then incubated overnight at 4 °C with an anti-βIII-tubulin antibody (1:1,000; T2200, Sigma-Aldrich) diluted in the blocking buffer. After washing with PBS, cells were incubated with an Alexa™ 488-conjugated anti-mouse IgG secondary antibody (Thermo Fisher Scientific) for 30 min at room temperature. Nuclei were counterstained with Hoechst 33,258 (H341, Dojindo, Kumamoto, Japan). Confocal images were obtained using a Nikon A1 microscope system (Nikon, Tokyo, Japan).

### 2.6 Payload release in pharmacological test

After 168 h of incubation of ADCs in cell culture medium, in the presence and absence of human iPSC-derived sensory neurons, payload concentrations in the medium and cell lysates were quantified using high-performance liquid chromatography-electrospray ionization tandem mass spectrometry (LC-ESI-MS/MS). During the 168-h exposure period, half-medium exchange with compound replenishment was performed after the 72-h measurement as described above. The incubated medium (50 μL) and cell samples were mixed with 200 μL of a methanol/acetonitrile mixture (1:1, v/v) containing propranolol as an internal standard (IS) and vortex-mixed thoroughly. After centrifugation, the supernatants were filtered and subjected to analysis.

LC-ESI-MS/MS analysis was performed using an ACQUITY Premier UPLC system coupled to a Xevo TQ Absolute mass spectrometer (Waters, Milford, MA, United States). Chromatographic separation was achieved using an ACQUITY UPLC BEH C18 column (1.7 μm, 2.1 mm × 100 mm; Waters) maintained at 40 °C with a flow rate of 0.4 mL/min. The mobile phases consisted of distilled water with 0.1% formic acid (A) and acetonitrile with 0.1% formic acid (B). The gradient elution program was as follows: isocratic elution at 5% B for 0.5 min; a linear gradient 5%–95% B from 0.5 to 3 min; isocratic elution at 95% B from 3 to 4 min; and re-equilibration at 5% B from 4.01 to 6 min.

Analytes were ionized by electrospray ionization in positive mode, and multiple reaction monitoring transitions were set at m/z 719.0 > 134.3 for MMAE, 552.7 > 84.3 for Lys-SMCC-DM1, 393.4 > 249.3 for SN-38, and 260.4 > 116.3 for propranolol (IS). Calibration curves were generated by linear regression analysis with a weighting factor of 1/x^2^ using the peak area ratio of analytes to the IS. The lower limits of quantitation (LLOQs) for MMAE, Lys-SMCC-DM1, and SN-38 were 0.0020, 0.060, and 0.0060 pmol/well, respectively, in cell lysate samples, and 0.15, 0.15, and 0.50 pmol/well, respectively, in medium samples. The corresponding quantitation ranges were 0.0020–60, 0.060–60, and 0.0060–600 pmol/well, respectively, in cell lysate samples, and 0.15–500, 0.15–500, and 0.50–5,000 pmol/well, respectively, in medium samples. The acceptance criterion for calibration sample accuracy was within ±20%, and all calibration samples met this criterion. For the analysis of measured payload levels, theoretical payload amounts were estimated using approximate or average ADC molecular weights, reported DAR values, ADC concentrations, and sampling volume, and measured/theoretical payload ratios were calculated by dividing the measured total payload amount by the theoretical payload amount. Recovery, matrix effects, processed-sample stability, freeze-thaw stability, stability of each payload and ADC in culture medium, and potential payload release or degradation during sample preparation and extraction were not evaluated in the present study. LC-MS/MS payload quantification data, including assay information and sample-level payload quantification data, are provided as a supplementary data file.

### 2.7 Statistical analysis

Longitudinal MEA electrophysiological and MEA-derived resistance data were analyzed using a repeated-measures modeling framework. The statistical models were applied to the normalized datasets shown in each figure. For Figure 1E, firing-rate values were normalized to the corresponding pre-treatment baseline (0-h value) within each treatment group. For Figures 1F–H, 2B, 3A–D, and Supplementary Figure S2, values were first normalized to the corresponding 0-h value within each treatment group and then normalized to the concurrent vehicle control at each time point. Because normalization to the 0-h value fixes the baseline values at 100% in these analyses, the 0-h values represent a fixed normalized baseline rather than variable baseline observations. Accordingly, *post hoc* comparisons versus 0 h were interpreted as tests of whether normalized post-treatment values differed from this fixed baseline value of 100%. For each compound and endpoint, a linear mixed-effects model was used with time point, dose, and their interaction as fixed effects, plate as a fixed covariate, and well identity as a random effect to account for repeated measurements from the same wells over time. For endpoints in which the linear mixed-effects model did not converge or resulted in a singular fit, a generalized estimating equation model with an exchangeable working correlation structure was used, with well identity treated as the clustering unit. Post hoc comparisons were performed to compare each post-treatment time point with the corresponding 0-h value within each dose group, with Dunnett-type adjustment for multiple comparisons. Statistical analyses were performed using Python (version 3.12) with the statsmodels package and R (http://www.r-project.org/). Results are presented as mean ± SEM. Statistical significance is indicated by the following: *p < 0.05, **p < 0.01, ***p < 0.001. Per-well MEA-derived electrophysiological and resistance datasets are provided as supplementary data files.

## 3 Results

### 3.1 Expression of ADC target antigens in human peripheral nerve tissue and iPSC-derived sensory neurons

To investigate whether ADC-induced neuronal functional changes may involve target-dependent mechanisms, we evaluated the expression of ADC target antigens in normal human peripheral nerve tissue and human iPSC-derived sensory neurons by immunostaining (Figure 4; Table 2). In human peripheral nerve tissue, no clear signals for CD30 (target of brentuximab vedotin), HER2 (target of trastuzumab emtansine), or Trop2 (target of sacituzumab govitecan) were observed in neurons or Schwann cells under the staining conditions used (Figures 4A,B). In contrast, granular HER2-positive signals were observed in vascular walls, and rare Trop2-positive signals were detected in stromal cells (Figures 4A,B). In human iPSC-derived sensory neurons, faint intracellular signals for CD30, HER2, and Trop2 were occasionally observed in a subset of cells. However, no clear membrane-associated antigen signals were observed on the plasma membrane or along neurites, which represent potential sites for ADC binding and internalization (Figure 4C). Collectively, these findings indicate that no clear membrane-associated antigen signals were observed in neurons within human peripheral nerve tissue or in human iPSC-derived sensory neurons under the staining conditions used, although limited signals were observed in non-neuronal cells within the peripheral nerve microenvironment.

**FIGURE 4:**
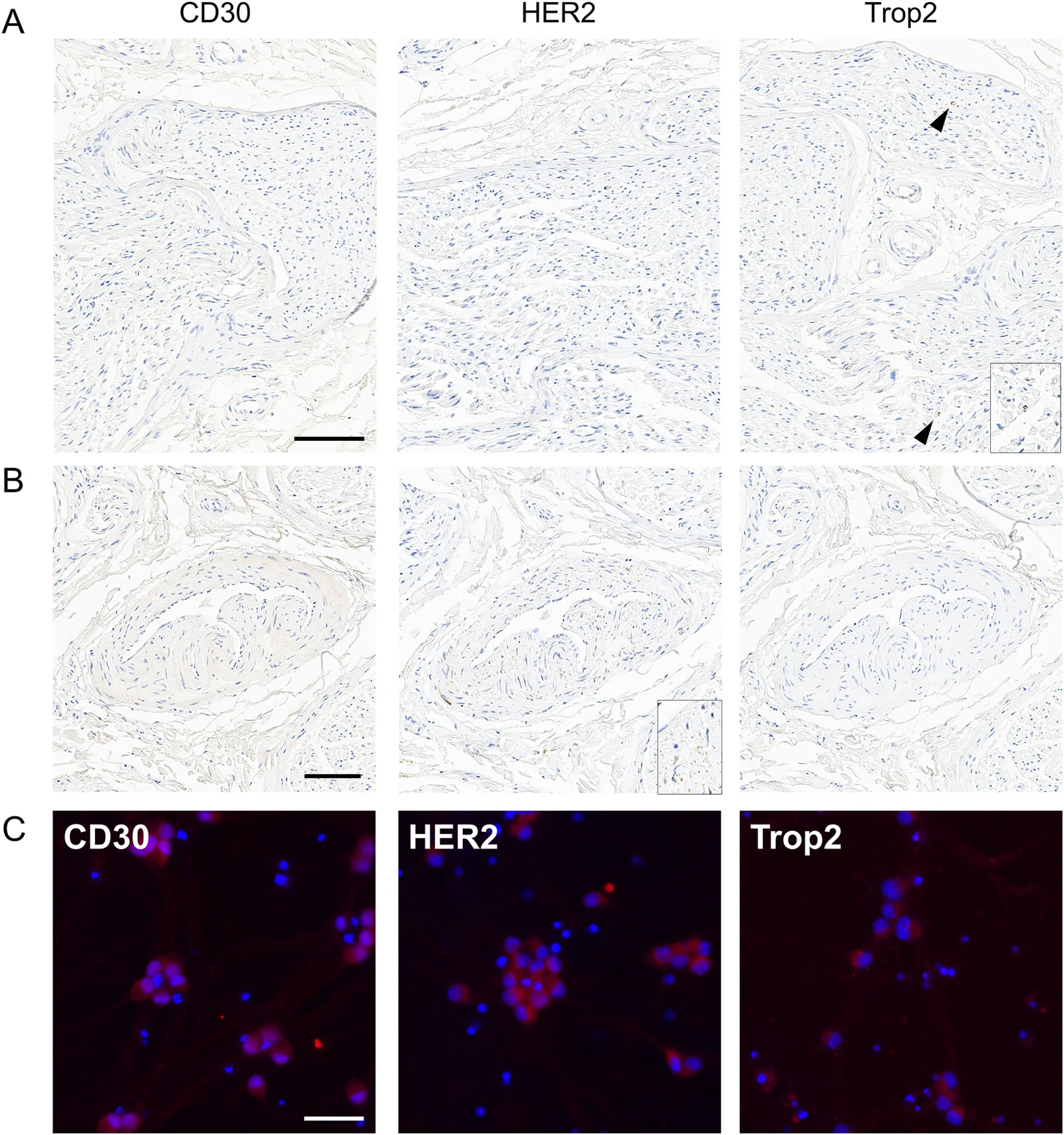
Expression of ADC target antigens in human peripheral nerve tissues and hiPSC-derived sensory neurons. **(A,B)** Representative immunostaining images of human peripheral nerve tissue sections stained for CD30, HER2, and Trop2. No detectable expression of these antigens was observed in neurons or Schwann cells. Occasional signals were detected in non-neuronal structures (e.g., vascular or stromal elements). Scale bars = 100 μm. **(C)** Immunofluorescence images of hiPSC-derived sensory neurons cultured for 4 weeks in vitro. No clear membrane-associated signals for CD30, HER2, and Trop2 (red) were observed on the neuronal cell surface or neurites. Nuclei are counterstained with DAPI (blue). Scale bar = 50 μm.

**TABLE 2:** Summary of expression of ADC target antigens in human peripheral nerve tissues and iPSC-derived sensory neurons.

ADC	Antigen	Human peripheral nerve tissues				Single cells (hiPSC-derived sensory neurons)
		Neuronal axon	Schwann cell	Interstitial cells	Blood vessels	
Brentuximab vedotin	CD30	-	-	-	-	Cytoplasm (+)
Trastuzumab emtansine	HER2	-	-	-	+	Cytoplasm (+)
Sacituzumab govitecan	Trop2	-	-	±	-	Cytoplasm (+, granular)

### 3.2 Functional assessment of human iPSC-derived sensory neurons using MEA

Approximately 4 weeks after seeding human iPSC-derived sensory neurons onto MEA plates, spontaneous neuronal activity was recorded using the Maestro Pro system (Axion BioSystems) (Figure 1A). Representative extracellular action potential waveforms recorded from a single electrode are shown in Figures 1B,C, and a representative raster plot across all electrodes within a well is shown in Figure 1D.

To evaluate the electrophysiological impact of representative ADC payloads, neuronal activity was recorded following treatment with MMAE, Lys-SMCC-DM1, and SN-38. The concentration ranges were selected based on literature-derived IC50 values from cancer cell-based cytotoxicity assays (European Medicines Agency, 2012; Perrone et al., 2020; Guerra and Alberti, 2022; Takeda Pharmaceutical Company Limited, 2023; Tomabechi et al., 2023; Chugai Pharmaceutical Co., Ltd., 2024) and are summarized in Table 1 and Supplementary Table S1. Because human iPSC-derived sensory neurons exhibited sparse and weakly synchronized spontaneous firing under the present conditions, electrophysiological analyses focused on spike-based parameters, including firing rate, mean spike amplitude, and coefficient of variation (CV) of spike amplitude. Recordings were obtained for 15 min at 0, 2, 24, 72, and 168 h across four concentration conditions. Responses to the vehicle control (DMSO) were also recorded. Figure 1E shows firing-rate changes normalized to the 0 h value within each treatment group. A gradual decrease in firing rate over time was observed across all conditions, including the DMSO control (Figure 1E). At the lowest concentration of SN-38, firing rates were significantly decreased at both 72 and 168 h. In contrast, higher concentrations showed significant increases at 168 h. At the highest concentration, this increase at 168 h followed a marked decrease observed at 72 h. To clarify payload-specific effects while minimizing the influence of time-dependent changes in vehicle-treated cultures, the data were additionally normalized to the corresponding DMSO control at each time point (Figures 1F–H). Based on the normalized data, a suppressive response characterized by reduced firing frequency was observed after 72 h for MMAE and Lys-SMCC-DM1, particularly at the highest concentration (Figure 1F). In contrast, SN-38 significantly increased firing rates at 168 h at all tested concentrations. Analysis of mean spike amplitude revealed a significant decrease at 168 h at the highest concentration of MMAE, while changes in Lys-SMCC-DM1 were less consistent across concentrations (Figure 1G). In contrast, SN-38 significantly increased mean spike amplitude at 168 h at all tested concentrations. For the CV of spike amplitude, significant decreases were observed at the highest concentrations of MMAE and Lys-SMCC-DM1, whereas SN-38 showed only limited changes (Figure 1H). Taken together, these results indicate that the tested payloads modulate neuronal electrophysiological activity in payload-specific patterns. Notably, SN-38 showed a distinct activity profile characterized by increased firing rates and mean spike amplitudes at 168 h, whereas MMAE and Lys-SMCC-DM1 primarily showed suppressive effects on firing activity, particularly at the highest concentration, suggesting differences in their functional impact on neuronal activity.

### 3.3 Effects of payloads on cell morphology and resistance in human iPSC-derived sensory neurons

We next evaluated the effects of each payload on cell morphology and resistance in human iPSC-derived sensory neurons. A heatmap of resistance values acquired concurrently with MEA recordings is shown in Figure 2A. Compared with the DMSO control, cells treated with MMAE and Lys-SMCC-DM1 showed a time-dependent increase in resistance levels. Similarly, an increase in resistance was observed with SN-38 at 168 h. To quantitatively assess these changes, the resistance values were first normalized to the corresponding 0-h value and then normalized to the corresponding DMSO control at each time point (Figure 2B). MMAE and Lys-SMCC-DM1 induced significant, time-dependent increases in resistance at higher concentrations. In contrast, SN-38 induced a significant increase in resistance at 168 h at all tested concentrations. Cellular morphology following MEA recording was further evaluated by βIII-tubulin immunostaining at 168 h (Figure 2C; Supplementary Figure S1). For MMAE, no obvious morphological alterations were observed at dose 3, whereas marked alterations in βIII-tubulin-positive neuronal structures, including neurite fragmentation and disruption of the neurite network, were observed at dose 4. A similar pattern was observed for Lys-SMCC-DM1, with neuronal morphology relatively preserved at dose 3 and marked structural alterations observed at dose 4. In contrast, no obvious morphological alterations were observed in SN-38-treated cells, even at the highest concentration (1 μg/mL). Taken together, these findings indicate that all tested payloads influence resistance under higher concentration conditions, although the associated morphological changes differed between compounds. Notably, the marked structural alterations observed at dose 4 of MMAE and Lys-SMCC-DM1 coincided with the suppression of neuronal activity shown in Figure 1F.

To further assess the reproducibility of the electrophysiological responses, an additional independent experiment was performed using a different neuronal lot (Supplementary Figure S2). MMAE significantly reduced spike rate at 10 ng/mL at 72 and 168 h, consistent with the suppressive response observed in the main experiment (Figure 1F). In contrast, SN-38 significantly increased spike rate at 1 and 10 ng/mL at later time points, with significant increases observed at 72 and 168 h, consistent with the activity-enhancing response observed in the main experiment. These findings indicate that the distinct directions of the electrophysiological responses to MMAE and SN-38 were also observed in an independent experiment using a different neuronal lot.

### 3.4 Effects of ADCs on neuronal electrophysiological activity and resistance

As shown in Figures 1, 2, the major ADC payloads (MMAE, Lys-SMCC-DM1, and SN-38) displayed distinct functional profiles in human iPSC-derived sensory neurons. We next evaluated the effects of ADCs containing these payloads on neuronal function using the concentration ranges selected for each ADC in this study (Table 1).

Human iPSC-derived sensory neurons were treated with brentuximab vedotin, trastuzumab emtansine, or sacituzumab govitecan, and changes in electrophysiological activity and resistance were analyzed (Figure 3). Each parameter was first normalized to the corresponding 0-h value and then normalized to the corresponding DMSO control at each time point. Brentuximab vedotin and trastuzumab emtansine did not induce significant changes in firing rate across the tested concentration range (Figure 3A). Although sporadic significant changes were observed in mean spike amplitude and the CV of spike amplitude, these changes were limited and did not show a consistent dose- or time-dependent pattern (Figures 3B,C). Similarly, only limited and sporadic significant changes in resistance were observed for brentuximab vedotin and trastuzumab emtansine, without a consistent concentration- or time-dependent pattern (Figure 3D). These findings contrast with the suppressive effects on neuronal activity and the increases in resistance observed with the corresponding free payloads (MMAE and Lys-SMCC-DM1) (Figures 1, 2), suggesting that the prominent electrophysiological and resistance responses observed with free-payload exposure were largely not reproduced following treatment with the corresponding ADCs under the present experimental conditions. In contrast, sacituzumab govitecan exhibited a distinct response profile. At most of the tested concentrations, sacituzumab govitecan significantly increased firing rate at 168 h (Figure 3A), consistent with the increased neuronal activity observed with the SN-38 payload (Figure 1F). While only limited significant changes were observed in the CV of spike amplitude, both mean spike amplitude and resistance showed significant increases mainly at 168 h, particularly at higher concentrations (Figures 3B–D). This increase in resistance was also consistent with the response observed for SN-38 alone (Figure 2B). Taken together, these results indicate that brentuximab vedotin and trastuzumab emtansine have limited detectable effects on neuronal function in this MEA model, whereas sacituzumab govitecan showed functional effects on neuronal activity similar to those observed with its payload, SN-38.

### 3.5 Payload measurements following ADC exposure

To obtain endpoint payload measurements relevant to interpreting the SN-38-like neuronal activity observed with sacituzumab govitecan, cells and culture supernatants were collected after completion of the MEA recordings, and payload levels associated with each ADC were quantified by LC-MS/MS. The amount of payload detected in the culture medium, as well as the payload detected in cell lysates, were quantified under conditions with ADCs in medium only [Cell (−)] and in the presence of cells [Cell (+)]. In both Cell (−) and Cell (+) conditions, extracellular payload levels from brentuximab vedotin and trastuzumab emtansine were below or close to the lower limit of quantitation in the medium (Supplementary Figure S3A). In contrast, SN-38 associated with sacituzumab govitecan was detected in the medium under both conditions and showed a concentration-dependent increase (Supplementary Figure S3A). Consistently, intracellular analysis revealed detectable SN-38 only in sacituzumab govitecan-treated samples, whereas payloads from the other ADCs remained below the lower limit of quantitation (BQL) (Supplementary Figure S3B). When extracellular and intracellular payload amounts were combined to calculate measured total payload per well, sacituzumab govitecan showed the highest measured total payload values under the concentration conditions used in this study (Figure 5A). To examine potential cell-dependent differences in measured payload levels, differences between Cell (+) and Cell (−) conditions were evaluated. Sacituzumab govitecan showed a slight increase in payload levels under Cell (+) conditions at concentrations of 10 μg/mL or lower, whereas at 100 μg/mL, higher payload levels were observed under Cell (−) conditions (Figure 5B). To further evaluate measured payload levels under the concentration range selected for each ADC, we assessed the measured total payload amount at the highest concentration tested for each ADC relative to the theoretical payload amount (Table 3; Supplementary Table S2). Because half-medium exchange with compound replenishment was performed after the 72-h recording and the removed medium was not analyzed, the payload values measured at 168 h represent endpoint measurements rather than cumulative release over the entire 168-h exposure period. Therefore, we calculated the ratio of measured total payload at 168 h to the theoretical payload amount estimated from the ADC concentration, approximate or average ADC molecular weight, and DAR values. This analysis revealed condition-dependent differences in measured/theoretical payload ratios among the ADCs tested. Sacituzumab govitecan showed a measured/theoretical payload ratio of 94.6% at 168 h, whereas the ratios for brentuximab vedotin and trastuzumab emtansine were both below 0.2%. Taken together, these findings indicate that, under the concentration conditions used in this study, sacituzumab govitecan exhibited higher measured payload levels at the 168-h endpoint than brentuximab vedotin and trastuzumab emtansine.

**FIGURE 5:**
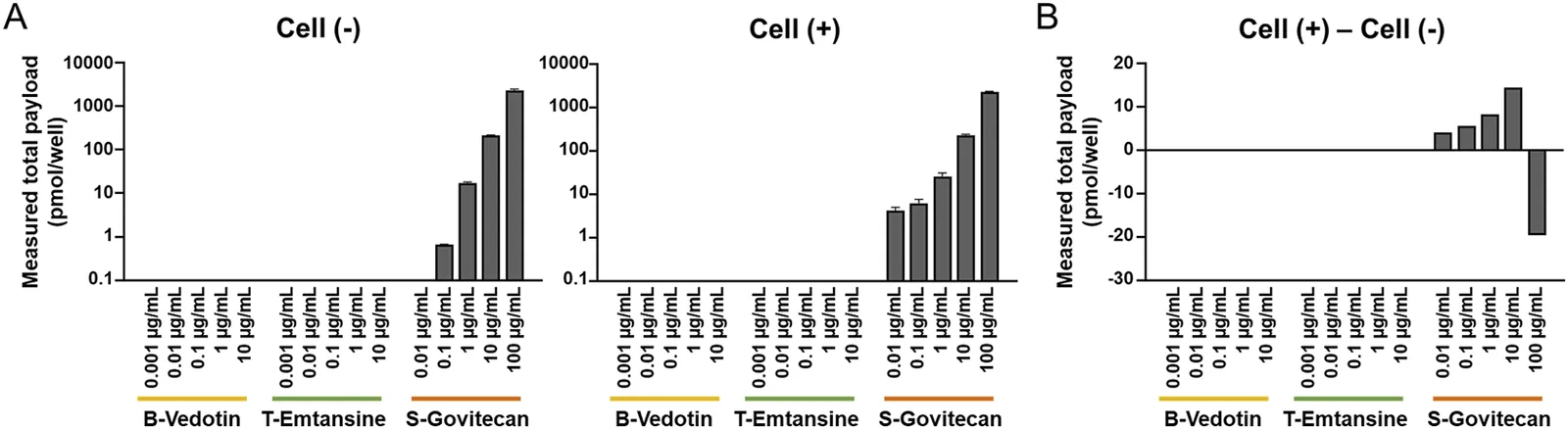
Measured total payload levels following ADC exposure. **(A)** Total amount of measured payload per well, calculated as the sum of payload detected in the culture supernatant and cell lysate fractions at 168 h post-treatment with brentuximab vedotin, trastuzumab emtansine, or sacituzumab govitecan under five concentration conditions. Cell-containing [Cell (+)] samples were collected following MEA measurements, whereas cell-free [Cell (−)] samples were processed in parallel to assess spontaneous, cell-independent payload release. **(B)** Comparison of mean measured total payload levels between Cell (−) and Cell (+) conditions. Data are presented as mean ± SEM (n = 6 wells for Cell (+) and n = 3 wells for Cell (−) per condition).

**TABLE 3:** Measured payload levels and measured/theoretical payload ratios at the highest ADC concentration.

ADC	Molecular weight	DAR	ADC concentration (ng/mL)	ADC concentration (nmol/L)	Conjugated payload concentration (nmol/L)	Theoretical payload amount (pmol/well)	Measured total payload at 168 h (pmol/well)	Measured/theoretical payload ratio (%)
Brentuximab vedotin	153,000	4.0	10,000	65	261	131	0.016	0.01
Trastuzumab emtansine	151,000	3.5	10,000	66	232	116	<0.21	<0.18
Sacituzumab govitecan	157,364	7.6	100,000	635	4,830	2,415	2,285	94.6

Theoretical payload amounts were calculated based on ADC, concentration, sampling volume, approximate or average ADC, molecular weight, and reported DAR values. Measured total payload indicates the sum of medium and cell lysate payload amounts at 168 h. The measured/theoretical payload ratio was calculated by dividing the measured total payload amount at 168 h by the theoretical payload amount. Half-medium exchange with compound replenishment was performed after the 72-h recording; the removed medium was not analyzed. Measured payload values at 168 h therefore represent endpoint measurements, not cumulative release over the entire 168-h exposure period. ADC, antibody-drug conjugate; DAR, drug-to-antibody ratio.

## 4 Discussion

Antibody-drug conjugates (ADCs) have demonstrated significant clinical utility in cancer therapy; however, adverse events—particularly peripheral neuropathy—remain a major challenge that often limits treatment continuity. Historically, ADC-induced neurotoxicity has been discussed primarily in relation to the pharmacological properties of payloads and linker stability; however, evaluations based on electrophysiological functional changes remain limited. In this study, we employed a microelectrode array (MEA)-based electrophysiological approach using human iPSC-derived sensory neurons to evaluate the effects of representative payloads (MMAE, DM1, and SN-38) and their corresponding ADCs on neuronal function. Furthermore, by integrating morphological analysis, resistance measurements, and quantitative assessment of released payloads obtained from the same cultures following MEA recordings, we performed an integrated evaluation of the effects of both free payloads and ADCs.

Using immunostaining, we observed no clear plasma membrane-associated signals for CD30, HER2, and Trop2 in neurons within human peripheral nerve tissue or in human iPSC-derived sensory neurons under the staining condition used (Figure 4; Table 2). ADC-induced toxicity has traditionally been discussed on the premise of binding to target antigens expressed on the surfaces of tumor cells, followed by internalization (Mahalingaiah et al., 2019; Ashman et al., 2022). However, because the present qualitative immunostaining analysis was not designed to rigorously establish the absence of cell-surface target antigen expression, these findings should be interpreted cautiously. Nevertheless, the neuronal activity changes observed in this model are consistent with the possibility that mechanisms not requiring detectable target antigen expression contributed to the observed responses. Taken together, our findings support consideration of possible target antigen-independent effects in the *in vitro* evaluation of ADC-associated neuronal functional changes (Mahalingaiah et al., 2019; Fu et al., 2023; Chen et al., 2024).

Our MEA recordings using free payloads revealed that the distinct pharmacological properties of each compound were reflected in the electrophysiological profiles of human iPSC-derived sensory neurons (Figure 1). Although both MMAE and Lys-SMCC-DM1 function as microtubule inhibitors, they differ substantially in their physicochemical properties. MMAE is characterized by high hydrophobicity and membrane permeability, allowing it to diffuse into neighboring cells and exert a bystander effect after release from ADCs (Okeley et al., 2010; Li et al., 2016). In contrast, Lys-SMCC-DM1—an active metabolite in which the maytansinoid derivative DM1 is conjugated to a lysine residue via a non-cleavable SMCC linker—is relatively hydrophilic and has been reported to exhibit very low membrane permeability (Kovtun et al., 2006; Erickson and Lambert, 2012; Ogitani et al., 2016). In this study, to approximate the major active forms released following ADC processing *in vivo*, MMAE was evaluated as the free payload, whereas DM1 was assessed as the lysine-linked SMCC conjugate. As shown in Figure 1, both MMAE and Lys-SMCC-DM1 reduced firing rates at higher concentrations. In addition, MMAE decreased mean spike amplitude at the highest concentration. These electrophysiological changes, together with the morphological alterations consistent with cytoskeletal disruption, support a suppressive effect of microtubule-inhibiting payloads on neuronal activity in this model. Notably, the reduction in firing activity was not accompanied by a decrease in resistance measured concurrently using the impedance-based MEA Viability assay; instead, resistance increased at higher concentrations of MMAE and Lys-SMCC-DM1 (Figure 2). The absence of decreased resistance argues against gross cell loss or detachment as the sole explanation for the reduced firing activity. However, βIII-tubulin staining revealed marked structural alterations at dose 4 of both MMAE and Lys-SMCC-DM1, indicating that preserved resistance does not exclude neuronal structural damage at these concentrations. Thus, the electrophysiological suppression observed at the highest concentrations may have been influenced, at least in part, by structural damage and potentially associated cytotoxic effects. In contrast, at MMAE dose 3, firing frequency was significantly reduced at 72 h, whereas no obvious morphological alteration was observed at the 168-h endpoint (Figures 1, 2). Because the morphological assessment was performed only at the 168-h endpoint, cytotoxicity at 72 h cannot be directly excluded. Nevertheless, the transient electrophysiological suppression observed at dose 3 was not associated with persistent overt structural damage at 168 h. Importantly, the impedance-based MEA Viability measurement and qualitative morphological assessment do not substitute for a direct quantitative cell-viability or cytotoxicity assay; therefore, subtle cytotoxic effects cannot be excluded, and an IC20 for these sensory neurons could not be determined in the present study. Therefore, these findings should be interpreted as electrophysiological and morphological alterations under the present experimental conditions, rather than as direct evidence of cytotoxicity or neurotoxicity. Notably, no marked functional differences were observed between MMAE and Lys-SMCC-DM1 under the present experimental conditions. This observation suggests that the suppression of neuronal activity in this model may be more closely related to their shared mechanism of microtubule disruption than to differences in membrane permeability. These findings are consistent with the high incidence of peripheral neuropathy reported for ADCs containing these payloads and may reflect impaired axonal transport and neuronal signaling associated with microtubule disruption (Chen et al., 2017; Chen et al., 2024; Fu et al., 2023).

In contrast to MMAE and Lys-SMCC-DM1, the topoisomerase I inhibitor SN-38 exhibited an opposing pattern of neuronal activity changes (Figure 1). SN-38 exerts cytotoxic effects by inhibiting the transient cleavage-religation processes of DNA during replication. Clinically, its primary dose-limiting toxicities are hematological (notably neutropenia) and gastrointestinal (Ocean et al., 2017; Schöffski et al., 2024; Ballestín et al., 2025). While the incidence of peripheral neuropathy is generally lower with SN-38-based ADCs than with those utilizing microtubule inhibitors, peripheral neuropathy has nevertheless been reported in approximately 20% of cases (U.S. Food and Drug Administration, 2020). These clinical observations suggest that the impact of SN-38 on the peripheral nervous system is not negligible and may differ mechanistically from that of microtubule inhibitors (Fu et al., 2023; Chen et al., 2024; Lesperance et al., 2025). In the present study, SN-38 was associated with increased neuronal activity, as indicated by elevated firing rates and mean spike amplitudes, without obvious morphological disruption across the tested concentrations (Figures 1, 2; Supplementary Figure S1). Moreover, the electrophysiological enhancement was not accompanied by a decrease in resistance; rather, resistance was increased at 168 h (Figure 2). These findings suggest that, unlike the structural alterations observed after microtubule-inhibitor treatment, SN-38 may modulate neuronal excitability. It has been reported that DNA damage responses, oxidative stress, and alterations in intracellular calcium homeostasis associated with topoisomerase I inhibition can influence neuronal excitability; such mechanisms may contribute to the functional changes observed with SN-38 (Chatterjee et al., 2001; Dai et al., 2014; Andres et al., 2022; Son et al., 2022; Lee, 2024). In addition, changes in neuronal density or network organization may also influence overall activity patterns, and therefore the observed electrophysiological changes may reflect not only alterations in single-cell excitability but also network-level effects. The SN-38-associated increase in neuronal activity observed in this study may thus represent functional modulation that was not accompanied by overt structural damage or a reduction in resistance. Importantly, increased firing activity alone is not sufficient to establish neurotoxicity, particularly because additional toxicity- and mechanism-related endpoints, such as cell viability, apoptosis, DNA damage, oxidative stress, calcium dynamics, or ion-channel activity, were not assessed in this study. Further studies incorporating these endpoints will be required to determine whether the observed increased neuronal activity reflects neuronal toxicity or non-toxic functional modulation and to clarify the underlying molecular mechanisms.

Our combined analysis of MEA recordings and payload quantification suggests that differences in measured payload levels may be associated with ADC-associated neuronal functional changes under the present experimental conditions (Figures 3, 5). Brentuximab vedotin and trastuzumab emtansine exhibited very low measured/theoretical payload ratios at the 168-h endpoint, and only limited detectable effects on neuronal activity were observed under the present experimental conditions, in contrast to the effects seen with their respective free payloads (Figures 1, 3, 5; Table 3). These findings are consistent with the interpretation that conjugation within ADCs may limit the direct impact of payloads on neuronal function under the present experimental conditions. However, the absence of detectable effects in this assay does not necessarily indicate that these ADCs lack neuronal liability. Rather, these results indicate that direct functional effects on isolated hiPSC-derived sensory neurons were not detected within the 168-h monoculture system used in this study, which may be related to the low measured payload levels under the present culture conditions. These findings should be interpreted with consideration of potential contributions from cumulative exposure, tissue pharmacokinetics, and interactions with peripheral nerve microenvironmental components. Furthermore, the low measured/theoretical payload ratios observed in this study are broadly consistent with clinical pharmacokinetic reports showing low circulating free payload exposure for these ADCs (Girish et al., 2012; Han et al., 2013). In contrast, under the concentration conditions used in this study, sacituzumab govitecan showed a high measured/theoretical payload ratio at the 168-h endpoint, including in the absence of cells, and was associated with neuronal activity changes similar to those observed with the free payload (Figures 3, 5; Table 3). The measured SN-38 concentration in the culture medium under Cell (+) conditions reached 4,426 nmol/L at the highest sacituzumab govitecan concentration, which was approximately 15-fold higher than the reported human Cmax of unconjugated SN-38 (298.2 nmol/L, 117 ng/mL) based on the FDA Multi-Discipline Review (U.S. Food and Drug Administration, 2020). These differences warrant cautious interpretation when extrapolating the present *in vitro* findings to *in vivo* neurotoxicity risk, clinical safety margins, or therapeutic windows. The high measured SN-38 level observed following sacituzumab govitecan exposure may be related, at least in part, to its hydrolytically sensitive linker structure, which has been designed to permit payload release, as well as to factors specific to the present *in vitro* culture conditions. For example, esterase-like activity at or near the cell surface, local pH variations, or interactions with the plasma membrane may facilitate SN-38 release even at lower concentrations. Consistent with this, SN-38 was detectable under cell-present conditions at low concentrations where it remained below the detection limit in the absence of cells, suggesting that the cellular environment may influence payload release behavior (Figure 5B and Supplementary Figure S3A). At the highest concentration, the total payload per well was lower under cell-present conditions than under cell-free conditions (Figure 5B). This observation suggests that released SN-38 may undergo cellular uptake, binding to intracellular components, or metabolic modification, thereby reducing the measurable fraction of the payload. These dynamics are consistent with the electrophysiological changes observed under the same conditions. Taken together, these findings are consistent with the possibility that SN-38 detected following sacituzumab govitecan exposure contributes to the observed neuronal functional changes. However, a direct causal relationship between the measured SN-38 levels and the electrophysiological effects of sacituzumab govitecan remains to be established. Further studies using additional antibody/linker-related controls and direct comparisons with free SN-38 at concentrations equivalent to the measured SN-38 levels would help strengthen this mechanistic interpretation.

In the present study, resistance at 41.5 kHz, the endpoint reported by the impedance-based MEA Viability measurement, was observed to increase under high-concentration conditions across all tested payloads (Figures 2A,B). This increase may reflect a combination of factors, including changes in cell density near the electrodes associated with somatic shrinkage and cytoskeletal disruption, the accumulation of cellular debris, and characteristics inherent to the MEA measurement system. Indeed, for the microtubule inhibitors MMAE and Lys-SMCC-DM1, marked morphological alterations such as somatic shrinkage and neurite fragmentation were observed (Figure 2C), and these structural changes may have contributed to the increased resistance. Notably, an increase in resistance was also observed in the SN-38-treated group despite the absence of obvious cytoskeletal disruption (Figure 2). This observation suggests that resistance changes do not necessarily reflect cytoskeletal damage alone; rather, multiple cellular responses, including alterations in cell adhesion, membrane properties, or subtle intracellular changes, may contribute to the observed electrical changes. Previous studies using cultured rat dorsal root ganglion (DRG) neurons have reported impedance-based resistance measurements for evaluating peripheral neurotoxicity (Han et al., 2023). In the present study, resistance increases were observed primarily under conditions in which neuronal functional changes were also detected by MEA, suggesting that MEA-derived resistance may serve as a complementary functional readout of cellular responses associated with neuronal changes. However, resistance changes alone do not establish a specific neurotoxicity mechanism, and further studies combining morphological and functional analyses will be required.

In this study, we showed that MEA analysis using human iPSC-derived sensory neurons can detect neuronal functional alterations induced by major ADC payloads, and that sacituzumab govitecan induces neuronal functional changes similar to those observed with the payload alone. In contrast, the inhibitory effects observed with MMAE and Lys-SMCC-DM1 alone were not detected for brentuximab vedotin or trastuzumab emtansine under the present experimental conditions. This difference may be attributable, at least in part, to the lack of target antigen expression on the cell surface of human iPSC-derived sensory neurons, as well as the extremely low levels of payload release under the culture conditions. Thus, this system primarily captures direct neuronal functional changes associated with released payloads, rather than effects mediated through antibody-dependent internalization. For ADCs with high linker stability, effects on neuronal activity may be less readily detectable under short-term *in vitro* conditions. However, peripheral neuropathy associated with brentuximab vedotin and trastuzumab emtansine has been reported clinically, suggesting that these clinical neurotoxicities may not be fully explained by direct effects on neurons alone. Other factors, including cumulative exposure and indirect effects mediated by the peripheral nerve microenvironment, may also contribute *in vivo*. These limitations are discussed below. Therefore, while the present model does not recapitulate the full spectrum of ADC-induced peripheral neurotoxicity, it provides a focused analytical approach for evaluating payload release behavior and its direct functional impact on neurons. These considerations are further addressed in the limitations below.

Several limitations of the present study should be acknowledged. In this study, we employed a simplified monoculture system of human iPSC-derived sensory neurons to focus on direct functional responses of sensory neurons to ADCs and their payloads under *in vitro* conditions. According to a published transcriptomic analysis of this cell model, non-sensory neuronal cell types, including glial cells, fibroblasts, and immune cells, were not identified in the present model. Accordingly, although this system is suitable for evaluating direct functional responses of sensory neurons, it does not reproduce interactions with surrounding cell types or other components of the peripheral nerve microenvironment, including Schwann cells, satellite glial cells, immune cells, vascular components, and blood–nerve barrier-related pharmacokinetic processes. Therefore, the present findings should be interpreted as direct neuronal responses under simplified *in vitro* conditions, and further refinement of the experimental system will be necessary to better reproduce peripheral neurotoxicity *in vivo*. Furthermore, the detailed composition of the proprietary culture medium used in this study was not disclosed by the supplier. Therefore, potential effects of medium components on ADC integrity and measured payload levels cannot be completely excluded. In addition, LC-MS/MS-based payload quantification was performed only at the 168-h endpoint and therefore does not establish a temporal relationship between measured payload levels and the observed neuronal functional changes. Potential payload release or degradation during sample preparation and extraction was not specifically evaluated, and ADC integrity and linker stability were not directly assessed in the present study. Accordingly, the measured payload values should be interpreted as endpoint measurements under the present experimental conditions rather than as a formal assessment of ADC linker stability or direct evidence that payload release caused the observed neuronal functional changes.

Because ADCs are heterogeneous conjugates generally characterized by approximate molecular weights and average drug-to-antibody ratios, ADC concentrations in this study were expressed as mass concentrations to facilitate compound preparation and reproducibility. Consequently, the ADCs were not compared under strictly equivalent molar or equipayload conditions, which remains a limitation of the present study. In addition, sacituzumab govitecan was tested at a higher maximum mass concentration than brentuximab vedotin and trastuzumab emtansine, and therefore comparisons among ADCs should be interpreted as condition-specific comparisons rather than direct equivalent-exposure comparisons. Future studies incorporating molar- or equipayload-based comparisons, including direct comparison between ADCs and their corresponding free payloads at concentrations equivalent to the measured payload levels, would further clarify the relationship between ADC exposure, payload release, and neuronal functional changes. An additional independent experiment using a different neuronal lot showed electrophysiological response patterns to MMAE and SN-38 that were consistent with those observed in the main experiment (Supplementary Figure S2). However, such independent validation was not performed for all tested payloads and ADCs, and the multiple wells analyzed within each experiment should be regarded as within-experiment replicates rather than independent biological replicates. Therefore, the present findings should still be interpreted as a proof-of-concept evaluation of this integrated *in vitro* platform, and further validation across independent experiments, neuronal lots, and culture batches will be required to confirm the reproducibility and generalizability of the findings. In addition, morphological assessment in the present study was primarily qualitative and based on representative βIII-tubulin staining, and quantitative characterization of neurite morphology and neuronal structure was not performed. Furthermore, although impedance-based MEA Viability measurements were performed concurrently with electrophysiological recordings, a direct quantitative cell-viability or cytotoxicity assay was not conducted; therefore, subtle cytotoxic effects cannot be excluded and neuronal IC20 values could not be determined. Future studies incorporating additional validation using known neuroactive and neurotoxic reference compounds, repeated exposure paradigms, and additional cellular components may further improve the translational relevance of this platform. Despite these limitations, the present *in vitro* platform may serve as a useful complementary approach for detecting neuronal functional alterations and comparing candidate ADCs during early-stage ADC development.

## Data Availability

The raw data supporting the conclusions of this article will be made available by the authors, without undue reservation.

## Supplementary material

Supplementary Table 1 Reported IC_50_ values used to guide concentration range selection. Reported IC_50_ values were collected from published *in vitro* proliferation or cytotoxicity assays and used as approximate reference values to guide the selection of broad logarithmic concentration ranges in this study. Because these assays were performed using cancer cell lines or non-neuronal assay systems rather than human iPSC-derived sensory neurons, the values were not intended to define neuronal toxicity thresholds or clinically relevant exposure levels. For SN-38, the IC_50_ value was reported for a cytotoxicity assay, but the cell line was not specified in the source.

Supplementary Table 2 Calculation of theoretical payload amounts across ADC concentration ranges. Theoretical payload amounts were calculated using ADC-specific approximate or average molecular weights, reported drug-to-antibody ratio (DAR) values, and a well volume of 500 μL. ADC amount was calculated from ADC concentration and well volume. These values were used as the basis for the measured/theoretical payload ratios shown in Table 3.

Supplementary Figure 1 βIII-tubulin morphology of hiPSC-derived sensory neurons following treatment with payloads. Representative βIII-tubulin immunofluorescence images of hiPSC-derived sensory neurons obtained at 168 h after treatment with MMAE, Lys-SMCC-DM1, or SN-38 at doses 1–4. A representative image of the DMSO-treated control is also shown. Scale bar = 200 μm.

Supplementary Figure 2 Electrophysiological responses to MMAE and SN-38 in an independent experiment using a different neuronal lot. Spike firing frequency following treatment with **(A)** MMAE and **(B)** SN-38 was evaluated in an independent experiment using a neuronal lot different from that used in the main experiment (Figure 1F). MMAE and SN-38 were tested at 0.01, 0.1, 1, and 10 ng/mL. Data were first normalized to the 0-h value within each treatment group and then normalized to the corresponding vehicle control at each time point. Measurements were obtained from 15 min recordings at 0, 2, 24, 72, and 168 h post-treatment (n = 6 wells per condition). Statistical analysis was performed using a linear mixed-effects model or generalized estimating equation model for repeated-measures data, followed by Dunnett-type adjusted comparisons versus the corresponding 0-h value within each dose group (**p* < 0.05, ***p* < 0.01, ****p* < 0.001). Data are presented as mean ± SEM.

Supplementary Figure 3 Extracellular and intracellular payload levels following ADC exposure. **(A)** Amount of extracellular payload detected in the culture supernatant under cell-free [Cell (−)] and cell-containing [Cell (+)] conditions following treatment with brentuximab vedotin, trastuzumab emtansine, or sacituzumab govitecan under five concentration conditions. Samples were collected at 168 h post-treatment; Cell (+) supernatants were collected following MEA measurements, whereas Cell (−) samples were processed in parallel to assess spontaneous, cell-independent payload release. **(B)** Intracellular payload levels quantified in hiPSC-derived sensory neurons under Cell (+) conditions following treatment with each ADC at 168 h post-treatment. Data are presented as mean ± SEM (n = 6 wells for Cell (+) and n = 3 wells for Cell (−) per condition).
